# Evaluation of the EPR Effect in the CAM-Model by Molecular Imaging with MRI and PET Using ^89^Zr-Labeled HSA

**DOI:** 10.3390/cancers15041126

**Published:** 2023-02-09

**Authors:** Colmar Hilbrig, Jessica Löffler, Gabriel Fischer, Ellen Scheidhauer, Christoph Solbach, Markus Huber-Lang, Ambros J. Beer, Volker Rasche, Gordon Winter

**Affiliations:** 1 Department of Nuclear Medicine, Ulm University Medical Center, 89081 Ulm, Germany; 2 Center for Translational Imaging, Core Facility Small Animal Imaging, Ulm University, 89081 Ulm, Germany; 3 Institute for Clinical and Experimental Trauma-Immunology, Ulm University Medical Center, 89081 Ulm, Germany; 4 Department of Internal Medicine, Ulm University Medical Center, 89081 Ulm, Germany

**Keywords:** CAM model, CAM, chick embryo, PET, MRI, barrier defects, HSA, EPR, mice

## Abstract

**Simple Summary:**

The CAM model is a promising alternative to murine models in terms of the 3Rs principles. However, its value for the noninvasive assessment of the biodistribution and accumulation of radiolabeled macromolecules by PET and MR imaging needs further evaluation. Thus, we analyzed the biodistribution and nonspecific tumor accumulation based on the EPR effect of ^89^Zr-labeled human serum albumin by PET and MRI in the xenograft CAM model. The results were correlated to in vivo results from the xenografted mouse model as the standard of reference. In both models, the xenografted TZM-bl and PC-3 tumors were visualized by PET imaging after 24 h. Furthermore, no significant differences were detected concerning the influx kinetics of ^89^Zr-labeled albumin into the two tumors. Therefore, the chicken model is a potential alternative to the animal model for initial PET studies on the characteristics of EPR-dependent target accumulation of radiolabeled macromolecules.

**Abstract:**

Mouse models are commonly used to study the biodistribution of novel radioligands, but alternative models corresponding to the 3Rs principles, such as the chorioallantoic membrane (CAM) model, are highly required. While there are promising data from the CAM model regarding target-specific radiolabeled compounds, its utility for assessing macromolecule biodistribution and analyzing the EPR effect remains to demonstrated. Using ^89^Zr-labeled human serum albumin, the accumulation of nontarget-specific macromolecules in CAM and mouse xenograft models was studied using PET and MRI. Therefore, the radioligand [^89^Zr]Zr-DFO-HSA was analyzed in both chicken embryos (n = 5) and SCID mice (n = 4), each with TZM-bl and PC-3 tumor entities. Dynamic PET and anatomical MRI, as well as ex vivo biodistribution analyses, were performed to assess ligand distribution over 24 h. Histological staining and autoradiography verified the intratumoral accumulation. The tumors were successfully visualized for CAM and mouse models by PET, and the albumin influx from the blood into the respective tumors did not differ significantly. The accumulation and retention of HSA in tumors due to the EPR effect was demonstrated for both models. These results highlight that the CAM model is a potential alternative to the mouse model for initial studies with novel radiolabeled macromolecules with respect to the 3Rs principles.

## 1. Introduction

Animal models provide initial insights into pharmacodynamics and pharmacokinetics, including the aspects of toxicity of novel pharmaceuticals [1]. However, concern for the welfare of laboratory animals has led to calls for animal welfare and regulation of the use of animals in research [2]. One of the first approaches was the publication of the 3Rs principles in the 1950s, which still serve as a guide for the use of animals in scientific research in many countries today [3]. The 3Rs principles state that animal testing should only be conducted under certain conditions: replacement of animal testing with alternative methods, reduction of the number of animals used, and refinement to minimize animal distress [4]. One possible alternative with regards to the aspects of replacement or reduction of murine animal models currently being developed is the chorioallantoic membrane (CAM) model. Initial studies on the accumulation of receptor-specific peptides have already been conducted [5,6,7], as have initial studies on drug delivery systems and antitumor treatment [8,9]. However, aspects of nontarget-specific accumulation of macromolecules in solid tumors are still insufficiently studied, which is of high relevance, as these may also influence the intratumoral accumulation of receptor-specific tracers or drugs [10]. When the increased permeability of blood vessels is coupled with the fact that lymphatic drainage within the tumor is impaired, it results in a phenomenon referred to as the enhanced permeability and retention (EPR) effect. The EPR effect and its influence on the nontarget-specific accumulation of macromolecules has been widely studied in solid tumors of mice xenografts and humans [11]. We decided to use zirconium-89 (^89^Zr)-labeled human serum albumin [^89^Zr]Zr-DFO-HSA as a representative for macromolecules.

Thus, in this study, the biodistribution and dynamics of nontarget-specific accumulation of [^89^Zr]Zr-DFO-HSA were analyzed by combined PET and MR imaging using tumor xenografts with different levels of perfusion and the expected EPR effect in the CAM model and in mice as the standard of reference.

## 2. Materials and Methods

### 2.1. Synthesis, Radiolabeling, and Stability

Chelator coupling to human serum albumin (HSA) (Sigma-Aldrich, St. Louis, MO, USA) was performed slightly modified in accordance to Vosjan et al. [12]. In brief, a 10 mM *p*-SCN-DFO (Macrocyclics Inc., Plano, TX, USA) solution was prepared in dry DMSO (Sigma-Aldrich, St. Louis, MO, USA), and 13 mg HSA was dissolved in 4 mL PBS and adjusted to pH = 9 with 0.1 M Na_2_CO_3_. A 3- to 4-fold molar excess (80 µL) of the *p*-SCN-DFO was added to the HSA solution. The mixture was incubated at 37°C for 4 h on an agitating heating block at 350 rpm. The resulting conjugate was purified using a centrifugal filter unit with a weight cut-off of 30 kDa (Amicon Ultra 30 K 2 mL Merk Millipore, Carrigtohill, Ireland). Protein concentration was determined using Coomassie Protein Assay Reagent (Thermo Fisher Scientific, Waltham, MA, USA) according to the manufacturer’s instructions on a Multiskan™ GO Microplate Spectrophotometer (Thermo Fisher Scientific, Waltham, MA, USA) at a wavelength of 595 nm. Radiolabeling of the DFO-HSA was performed using ^89^Zr^4+^, which was purchased as ^89^Zr(C_2_O_4_)_2_ from PerkinElmer Inc. (Waltham, MA, USA). With a half-life of 78.4 h [13], ^89^Zr was selected for the consecutive measurements directly after injection and 24 h post-injection. The purchased ^89^Zr was purified by exchanging the anion from oxalate to chloride via an anion exchange cartridge (Sep-Pak Accell Plus QMA Plus Light, Waters GmbH, Eschborn, Germany). The HCl-eluted (1M, Merck KGaA, Darmstadt, Germany) ^89^ZrCl_4_ solution was adjusted to a pH of 5–6 using 1 M Na_2_CO_3_ (Merck KGaA, Darmstadt, Germany). For radiolabeling, 2 and 8 MBq, respectively, of the ^89^ZrCl_4_ solution was added to 290 µg of the DFO-HSA. This results in a 70-fold molar excess of DFO-HSA for 2 MBq and a 17-fold molar excess for 8 MBq of the ^89^ZrCl_4_ solution. The volume was adjusted to a total volume of 620 µL, with 0.9% NaCl solution (B. Braun SE, Melsungen, Germany). For a radiochemical yield of at least 95%, radiolabeling was performed for 24 h at room temperature. Stability studies were performed after labeling of DFO-HSA was completed after 24 h by incubation, the radiotracer in human serum for 72 h at room temperature. To ensure that the complexed zirconium-89 with DFO was securely bound to the HSA and not free in solution, the solution was filtered through a 30 kDa filter (Amicon Ultra 30 K 0.5 mL Merk Millipore Carrigtwohill Ireland) by centrifugation according to the manufacturer’s instructions (Mikro 220R Hettich Zentrifugen, Tuttlingen, Germany) before each stability measurement. The resulting filtrate was used to determine the amount of free DFO present in the solution. Radiolabeling and the subsequent stability of [^89^Zr]Zr-DFO-HSA was confirmed by thin-layer chromatography separating [^89^Zr]Zr-DFO-HSA solution and filtrate (1 µL) on a TLC Silica gel 60 RP-18 F_254_s (Merck KGaA, Darmstadt, Germany). Citrate buffer (2 M, pH = 5.5) was used as the mobile phase. The TLC plate was incubated on an imaging plate (Fujifilm K.K. Tokyo, Japan) for 1 min. The screen was read out with a FLA-3000 Fluorescence Laser Imaging Scanner (Fujifilm, Minato, Japan), and the percentages of free and DFO-HSA-bound ^89^Zr were determined using AIDA image analysis software (Elysia-Raytest GmbH, Straubenhardt, Germany).

### 2.2. Preparation of Cell Culture

The HeLa cell line derivate TZM-bl (NIH AIDS Reagent Program, Germantown, MY, USA) [14] and the prostate carcinoma cell line PC-3 (ACC465, DSMZ, Braunschweig, Germany) [15] were used to establish tumor xenografts in the CAM model and in SCID mice, since both are thoroughly researched cancer cell lines exhibiting similar vascularity and growth of tumor volumes [16,17,18]. TZM-bl cell line-based xenograft, a HeLa cell derivative, grow to highly vascularized tumors with necrotic regions [19]. PC-3 tumor xenografts grow invasively and proliferate strongly [20], are less vascularized and have a hypoxic, as well as a necrotic, core [21]. Cell lines were cultured as described elsewhere [22]. Cell counting was performed using an improved Neubauer hemocytometer (C-chip, DHC-N01, NanoEnTek, Seoul, Republic of Korea).

### 2.3. Internalisation of [^89^Zr]Zr-DFO-HSA

To determine the uptake of radiolabeled HSA into PC-3 and TZM-bl, cells were incubated with [^89^Zr]Zr-DFO-HSA or ^89^Zr (control) for 1 h and 24 h. Therefor, 30,000 cells were seeded onto 12-well plates (Merck, KGaA, Darmstadt, Germany) 48 h prior to the experiment. Unspecific adsorption to the plate was determined using additional wells without cells. and 16 h prior to the experiment, the medium was changed to serum-free medium. [^89^Zr]Zr-DFO-HSA was prepared as described above (see Section 2.1). Every well received 262 kBq ^89^Zr (pH 6.5) or 244 kBq [^89^Zr]Zr-DFO-HSA. After 1 h/24 h (at 37 °C and 5 % CO_2_) the medium was removed. Each well was washed using 1 mL PBS (PAN-Biotech GmbH, Aidenbach, Germany), and afterwards, 50 mM glycine-HCl buffer pH 2.8 was added and incubated for 5 min. After removal of glycine-HCl and an additional wash with PBS, cells were lysed using 1 M NaOH (Merck KGaA, Darmstadt, Germany). Wells that did not contain cells were also treated with NaOH. All 4 fractions were transferred to γ-counter tubes, and activity concentration was determined using a Wizard^2^-Detector Gamma counter (PerkinElmer Inc., Waltham, MA, USA).

### 2.4. CAM Experiments

CAM experiments were performed using a slightly modified protocol according to the previously published method [6]. Briefly, chick embryos were incubated at 37.8 °C and 65% relative humidity, starting on embryonic development day (EDD) 0. A small hole was drilled into the eggshell on EDD2. The hole was expanded on EDD5, and a silicone double-ring was placed on the CAM. On EDD6, 0.8 × 10^6^ PC-3 or 1.5 × 10^6^ TZM-bl tumor cells were grafted onto the CAM. Therefore, the corresponding cell number was mixed with growth matrix (30%, *v*/*v*) and applied in a total volume of 45 µL per ring. Daily monitoring of tumor growth and embryo health was performed by visual inspection. MR and PET imaging was performed on EDD15 and EDD16. Chick embryos were cooled at 4 °C for 120 min before MRI measurement to avoid motion artifacts (according to the protocols of Bain et al. 2007 and Zuo et al. 2017 [23,24]).

For the biodistribution studies in the CAM model, a catheter with a 30G needle (B. Braun, Melsungen, Germany) was placed into a blood vessel of the chorioallantoic membrane. This method allows to monitor the biodistribution right from the start of the injection. Following catheterization, each egg was positioned in the PET scanner, and the application of 150 µL [^89^Zr]Zr-DFO-HSA ((0.50 ± 0.07) µg/mL) was performed immediately after the start of the measurement. An average activity of (1.79 ± 0.43) MBq (median dose 1.99 MBq) was injected. The whole egg and catheter and syringe were measured separately using a dose calibrator (CRC-12, Capintec, NJ, USA) to determine the successfully applied radioactivity (100 % injected activity (%IA)) for further quantification. A total of 5 chick embryos with tumors were selected for measurements.

### 2.5. Animal Studies

The biodistribution of the radioligand was analyzed in male immunodeficient CB17/lcr-Prkdc scid/Crl mice (SCID; n = 4; Charles River Laboratories, Sulzfeld, Germany). Such as in the CAM approach, tumor xenografts of the human cervix carcinoma cell line TZM-bl and the human prostate carcinoma cell line PC-3 were established by administration of 1 × 10^6^ cells subcutaneously into the subscapular regions (left = TZM-bl; right = PC-3) of the SCID mice. Both tumors were allowed to develop over a period of two weeks after injection with regular control using a caliper (VWR International, Radnor, PA, USA).

The mice were anesthetized using 1.5% isoflurane in pressured air/oxygen (80%/20%), and a catheter was placed in the tail vein for intravenous injection. The animals were first measured in the MRI and then transferred to the PET. Immediately after the start of the PET measurements, 150 µL of [^89^Zr]Zr-DFO-HSA (0.53 ± 0.05) µg/mL were injected via the catheter, with an average activity of (0.70 ± 0.14) MBq (median activity 0.69 MBq). During measurements and during transport, the animals were kept continuously anaesthetized. Five mice were prepared for the studies, from which one had to be excluded due to extravasation.

The studies were approved (ethical approval code 1375) by the Regional Council (Regierungspräsidium Tübingen, Baden-Württemberg, Germany) in compliance with German laboratory animal experimentation act and study procedures were in accordance with the European Communities Council Directive of 22 September 2010 (2010/63/EU). All applicable institutional and national guidelines for the care and use of animals were followed.

### 2.6. MRI and PET Measurements

For MRI and PET, the precooled chicken eggs were placed in a custom 3D-printed holder. The holder allows imaging in both modalities without changing the position of the egg. Imaging was done immediately after tracer injection and 24 h p.i.

MRI measurements were performed according to the protocols of Zuo et al. 2015 and 2017 [24,25]. Data were obtained using a 60 mm (CAM) or a 72 mm (mice) quadrature volume T/R resonator on an 11.7 T small-animal MRI system (Bruker BioSpec 117/16, Bruker Biospin, Ettlingen, Germany).

For the chicken eggs, a T1-weighted 3D Fast Low-Angle Shot (FLASH) sequence covering the entire chicken egg was acquired as an anatomic reference for the subsequent PET ligand biodistribution measurements. The scan parameters were TR/TE = 5/2 ms, matrix size = 400 × 400, in-plane resolution = 150 × 175 µm^2^, slice thickness = 175, no interlayer gap and NSA = 1. With 400 slices, the whole egg was covered, resulting in an acquisition time of 3 min. Further, a high-resolution T2-weighted Multislice Rapid Acquisition with Relaxation Enhancement (RARE) sequence was used to accurately assess the tumor volume, location, and structure. The scan parameters were TR/TE = 4320/45 ms, matrix size = 650 × 650, in-plane resolution = 77 × 91 µm^2^, slice thickness = 500 µm, no interlayer gap, RARE factor = 8, and NSA = 4. 30 slices were required to cover the entire tumor region, resulting in an acquisition time of 20 min.

Anatomic images of the mouse were obtained with a Multislice FLASH sequence with acquisition parameters as: TR/TE = 150/1.5 ms, flip angle FA = 15°, matrix size = 750 × 300, in-plane resolution = 100 × 133 µm^2^, slice thickness = 500 µm, and NSA = 12. Forty slices were acquired with coronal orientation.

To evaluate the biodistribution of [^89^Zr]Zr-DFO-HSA in chick embryos and mice, a dynamic 60 min PET scan was performed using a small-animal PET scanner (Focus120, Siemens Medical Solutions, Inc., Erlangen, Germany). Focus120 has a high spatial resolution (<1.3 mm) and high sensitivity (approximately 7%) with a 12 cm diameter bore and 7.6 cm axial length [26]. The obtained list mode files were processed to generate histograms (sinograms). For the first hour, a time series of 23 dynamic images in frames of 6 × 20 s, 7 × 60 s, and 10 × 300 s was generated, while, for the second scan (24h p.i.), a simplified histogram was applied, including 12 frames with 5 min each. Reconstructions were performed with OSEM3D/MAP using 4 OSEM2D, 2 OSEM3D, and 18 MAP iterations with a matrix of 256 × 256 and a zoom factor of 1.5. MRI and PET data were fused by automatic rigid superposition using the PMOD software tool (PMOD Technologies, Zurich, Switzerland).

Based on the MR images, tumor xenografts of TZM-bl and PC-3 were manually selected as volumes-of-interest (VOI), as well as the whole mouse and chicken embryo model and further organs of interest, including the heart, brain, and blood. As part of the analysis, decay correction to the injection time was applied. Time–activity curves (TAC) of the PET data (CAM n = 5, Mice n = 4) were compared using GraphPad Prism ver. 9.4.1 (GraphPad Software, San Diego, CA, USA). In addition, the activity concentration ratios for tumor-to-blood based on the PET and γ-counter were calculated by dividing the tumor activity concentrations by the temporally corresponding blood activity concentrations. The ratio was expressed as mean value and standard error of the mean (SEM) and plotted over time starting at 15 min p.i. The unidirectional inflow rate (K_in_) can be determined from the slope of the straight line over the entire measurement period of 25 h [27].

### 2.7. Ex Vivo Validation

Excised tumor xenografts were washed to reduce overestimation of accumulated activity due to blood spillage. Following a 1 min wash using PBS, the extracted tumors of the CAM model were analyzed by γ-counter COBRA II (PerkinElmer Inc, Waltham, MA, USA) to accurately quantify the accumulated radioactivity. Mouse tumors were also rinsed to remove blood from the extraction. Tumor volumes (mL) were determined for the CAM and mouse model based on the MR images, while, in addition, the tumor weight (g) was determined. Quantification of the radioactivity in the tumor was based on decay-corrected γ-counter data in relation to the total activity injected into the chicken egg or mouse (percent injected activity, (%IA)). These data were normalized to the MRI-derived tumor volume or tumor weight. For the determined activity concentrations in (%IA/mL) or (%IA/g), the mean value ± standard deviation and, additionally, the median are reported in the sections below.

### 2.8. Digital Autoradiography and Histopathological Analyses

Tumors were fixed overnight with 4% formaldehyde solution (Thermo Fisher Scientific, Waltham, MA, USA) in phosphate-buffered saline (PBS), pH 7.4.

Tissue was dehydrated and paraffin-embedded before preparation of 4 µm sections for IHC and 10 µm sections for digital autoradiography (DAR) and H&E using a rotary microtome (Leica JUNG RM2045, Wetzlar, Germany). To determine the activity distribution in the tumors itself, DAR was performed. Tumor sections were place on an imaging plate (Fujifilm K.K. Tokyo, Japan) screen and incubated in the dark for 30 days. After incubation, the screen was analyzed using a Fluorescence Laser Imaging Scanner (FLA-3000, Fujifilm, Minato, Japan). Subsequently, the sections were deparaffinized, and cell nuclei were stained with hematoxylin (Waldeck GmbH & Co. KG, Münster, Germany) for 10 min. Cell bodies were stained using a 2% eosin (Waldeck GmbH & Co. KG, Münster, Germany) solution in water for 2 min. Slides were mounted using Entellan^®^ (Merck KGaA, Darmstadt, Germany).

For IHC, consecutive slices were used. Since HSA is not an intracellular target, antigen retrieval was unnecessary, and quenching of the endogenous peroxidase activity was performed immediately after deparaffinization. Tissue sections were treated with 3% hydrogen peroxide for 15 min. Slides were washed with TBS-T buffer (Tween^®^ 20, 0.1%, Sigma-Aldrich, St. Louis, MO, USA). Subsequently, slides were blocked for 1 h using a blocking buffer consisting of Animal-Free Blocker (Newark, CA, USA) with 0.3 M glycine (Merck KGaA, Darmstadt, Germany) and 0.1% Tween 20. Anti-HSA antibody (Sigma-Aldrich, Merck KGaA; Darmstadt, Germany, catalog A6684, 1:1000) was incubated overnight at 4 °C. The next day, sections were washed and incubated with biotinylated second antibody (VECTASTAIN^®^ Universal Quick Kit, Vector Laboratories, Newark, CA, USA) for 1 h. After another washing step, sections were treated with the avidin/biotin-based VECTASTAIN^®^ Elite^®^ ABC Reagent (HRP) (Vector Laboratories, Newark, CA, USA) for 1 h. Afterwards, slides were washed again, and the HRP substrate, Vector^®^ DAB Peroxidase Substrate (Vector Laboratories, Newark, CA, USA), was added for 30 sec. DAB substrate turnover was stopped by washing with running tap water. Cell nuclei were counterstained with hematoxylin (Waldeck GmbH & Co. KG, Münster, Germany) for 1 min. Following, the slides were mounted using Entellan^®^ (Merck KGaA, Darmstadt, Germany).

Images of the section were captured using a BZ-X810 (Keyence, Neu-Isenburg, Germany) with 4x magnification lens for overview images and 10x magnification lens for close-up images.

### 2.9. Statistical Evaluation

Mann–Whitney test, Student’s *t*-test, and simple linear regression were performed using GraphPad Prism (ver. 9.4.1 for Windows, GraphPad Software, San Diego, CA, USA). Linear regression was conducted between 16 min p.i. and the end of the PET scan and checked for significant differences in the slopes. A *p*-value < 0.05 was assumed statistically significant.

## 3. Results

### 3.1. Radiochemical Stability and Internalization Studies of [^89^Zr]Zr-DFO-HSA

**Figure 1:**
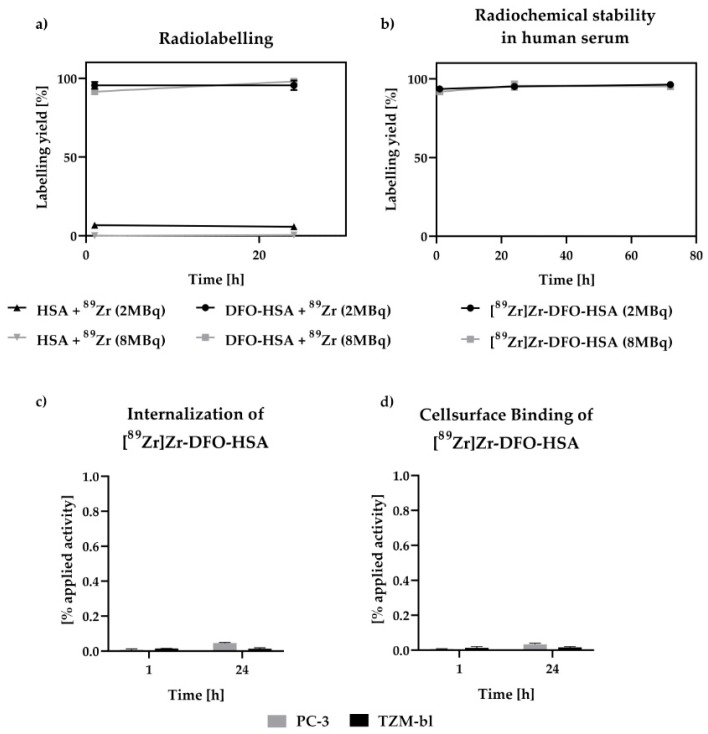
Depicted is the percentage of zirconium-89 complexed by DFO-HSA over a measurement period of 24 h. DFO-HSA was labeled with 2 or 8 MBq zirconium-89 respectively. A radiochemical yield greater than 95% was achieved after 1 h with 2 MBq zirconium-89, whereas this value was only achieved after 24 h with 8 MBq (**a**). Chelator-independent labeling of zirconium to HSA was neglectable. In both cases, the complex was stable in human serum for up to 72 h (**b**). The internalization process is evaluated over 24 h (**c**). No significant amount of [^89^Zr]Zr-DFO-HSA or zirconium-89 was detected within or bound (**d**) to either TZM-bl or PC-3 cells. By these results, it was demonstrated that DFO-HSA was successfully labeled with ^89^Zr and proven to be stable in human serum.

Already after one hour, a high labeling yield was achieved for 2 MBq of zirconium-89 ((98.38 ± 0.94) %) and only a slightly lower radiochemical yield for 8 MBq ((94.61 ± 0.58) %). After 24 h, for both applied activity amounts, most of the applied activity was bound to DFO-HSA (Figure 1a). Almost no unspecific binding of zirconium-89 to HSA occurs, since only a small fraction bound to HSA without DFO ((8.12 ± 2.15) % and (6.00 ± 0.39) %; Figure 1a). Over a period of 72 h, the stability of the complex was demonstrated, as no relevant amounts of zirconium-89 were detected in the filtrates (Figure 1b).

Neither relevant binding to the TZM-bl (0.02 ± 0.01) % applied activity (%AA) or PC-3 (0.03 ± 0.01) %AA cells nor internalization of [^89^Zr]Zr-DFO-HSA (TZM-bl (0.01 ± 0.01) %AA; PC-3 (0.05 ± 0.01) %AA) were detected over 24 h (Figure 1c,d; Tables S1–S3).

### 3.2. PET-MRI Analysis

**Figure 2:**
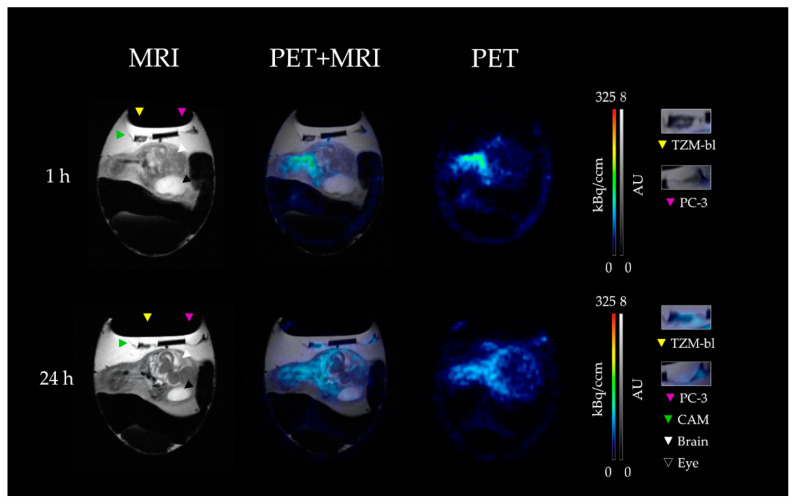
MR and PET images, including fusion images of the CAM model 1 h and 24 h after injection of [^89^Zr]Zr-DFO-HSA. In the T2-weighted sequence (**left**), the anatomy of the chick embryo and the tumors were clearly visible. The biodistribution of the radioligand could be observed by PET (**right**) and assigned to the respective organs in the corresponding fusion image (**middle**). Magnified images of the tumor region are separately depicted on the right. In the TZM-bl (left tumor, yellow arrow) and PC-3 (right tumor, magenta arrow) after one hour (**top row**), no accumulation of [^89^Zr]Zr-DFO-HSA could be detected, while after 24 h (**bottom row**), a clear accumulation was noted in both tumors. In the brain region (white arrow), no/only a low signal was observable after 1 h and 24 h. Thus, [^89^Zr]Zr-DFO-HSA displayed no unspecific accumulation in regions with intact barriers but in regions influenced by the EPR effect. The CAM (green arrow) and the eye of the embryo (black arrow) are additionally highlighted.

By visual inspection of the PET images over the measurement period (60 min p.i.), the highest activity concentration was observed in the blood for both the chick embryo model and the SCID mouse model (Figure 2; Figure S2; red and yellow color). However, no tumor nor brain signal was detected by PET imaging in neither model. 

**Figure 3:**
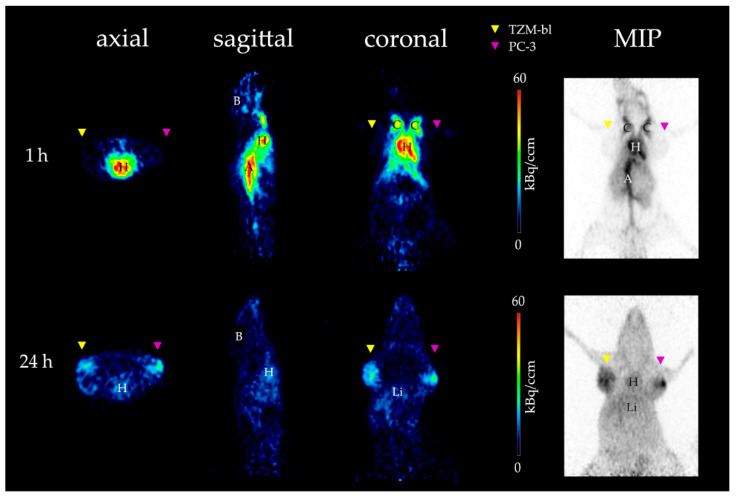
Image of [^89^Zr]Zr-DFO-HSA biodistribution in tumor-bearing mice by PET 1 h and 24 h p.i. Depicted are the three different section planes (axial, sagittal, and coronal) and the corresponding maximum intensity projection (**right**). After 1 h (**top row**), most of the radioactivity can be detected in the heart (H), aorta (A), and carotids (C). Within the two tumors (TZM-bl, yellow arrow, and PC-3, magenta arrow), as well as in the brain (B), little to no activity can be detected. After 24 h (**bottom row**), both tumors display a high activity level, whereas in the heart and liver (Li), only a moderate signal can be detected. Brain, joints, and kidneys are clear of radioactivity at 1 h and still after 24 h p.i., suggesting that there are no relevant amounts of free ^89^Zr due to the high stability of the compound. Thus, [^89^Zr]Zr-DFO-HSA accumulates only in regions affected by the EPR effect and shows similar results as in the CAM model.

After 24 h p.i., a lower activity concentration was observed in the blood, and both tumors (TZM-bl and PC-3) could then be visualized on the CAM of the chick embryo, as well as in the SCID mouse (Figure 2; Figure 3). Furthermore, no activity accumulation was observed in the brain, joints, and kidneys of the chick embryos and the SCID mice in PET imaging. However, the 24 h measurement of the brain region in the CAM model was noticeably affected by the adjacent meningeal blood vessels, which spilled over into the brain tissue and thus increased the measured activity concentration (Figure S3).

**Figure 4:**
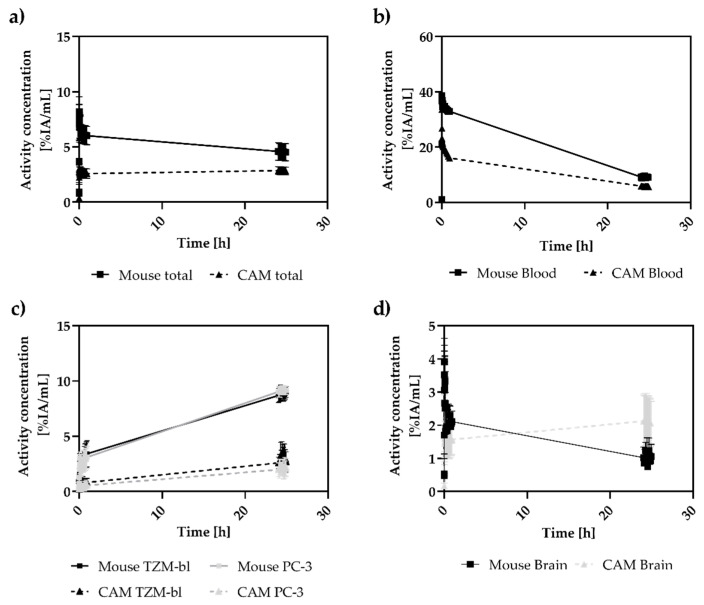
Depicted are the time–activity curves of both xenograft models: total activity in the different models (**a**), blood (**b**), TZM-bl and PC-3 tumors (**c**), and brain (**d**) over 24 h. In total, the activity concentration of the mouse tumors exceeds that of the CAM model (*p* < 0.001). In contrast, the activity concentrations in the avian brains surpassed those of the mice after 24 h (*p* < 0.001). However, within both xenograft systems, no differences in terms of accumulated activity in the different tumor entities can be detected.

Based on combined PET/MRI, a mean total activity concentration of (2.58 ± 0.42) %IA/mL was determined 60 min p.i. for the CAM model (Figure 4a; Tables S4–S8). This concentration remained in the same range with (2.79 ± 0.34) %IA/mL after 24 h p.i. (*p* = 0.095).

For the mouse model, a mean total activity concentration of (7.16 ± 1.48) %IA/mL was determined after the first scan, 60 min p.i. A significant lower activity concentration of (4.52 ± 0.67) %IA/mL was determined after 24 h p.i. (*p* < 0.001).

The time activity curves demonstrated similar pharmacokinetics for both models. For the CAM model immediately after tracer injection (70 s p.i.), the highest activity concentration was detected in the blood (heart VOI) with values of (23.62 ± 2.64) %IA/mL (Figure 4b). However, the concentration continuously decreased over time, with an average slope of m_Blood_ = −0.55 (%IA/mL)/h; R^2^ = 0.72. A significantly lower activity concentration of (5.15 ± 1.05) %IA/mL was detected after 24 h (*p* < 0.001).

The time–activity curves of the tumors supported the visual evaluation of the PET imaging (Figure 4c); a low tracer concentration was measured in the xenografts of TZM-bl ((1.20 ± 0.91) %IA/mL) and PC-3 ((0.60 ± 0.66) %IA/mL) 70 s after injection, followed by an increase within the following 24 h with a slope of m_TZM-bl_ = 0.08 (%IA/mL)/h; R^2^ = 0.86 and m_PC-3_ = 0.08 (%IA/mL)/h; R^2^ = 0.96, respectively, to a final activity concentration of (2.82 ± 0.97) %IA/mL (TZM-bl) and (2.62 ± 0.47) %IA/mL (PC-3). There were no significant differences between the two tumor entities in terms of the final activity concentration (*p* = 0.051). The initial measured activity concentration in the chick embryo brain region was determined with (1.56 ± 0.43) %IA/mL within the first hour (Figure 4d). Within the next 24 h, a significant increase was observed to a final activity concentration of (2.12 ± 0.68) %IA/mL (*p* < 0.001).

A similar pattern in the pharmacokinetics of the radiolabeled DFO-HSA was found in the SCID mice xenograft model. A high initial activity concentration was detected in the blood pool based on the heart VOI ((38.54 ± 5.94) %IA/mL; 70 s p.i.), which decreased continuously after 24 h to (9.03 ± 0.64) %IA/mL with an average decline of m = 0.92 (%IA/mL)/h; R^2^ = 0.63 (Figure 4b).

For the tumor xenografts of TZM-bl and PC-3, an activity concentration of (2.29 ± 0.57) %IA/mL and (2.31 ± 0.73) %IA/mL was determined at the baseline, respectively (Figure 4c). The activity concentrations progressively increased over the measurement period, with an average slope of m_TZM-bl_ = 0.27 (%IA/mL)/h; R^2^ = 0.97 and at m_PC-3_ = 0.28 (%IA/mL)/h; R^2^ = 0.98. A final activity concentration of (8.99 ± 0.43) %IA/mL (*p* < 0.001) for TZM-bl and (9.16 ± 0.32) %IA/mL; (*p* < 0.001) for PC-3 was detected after 24 h. Again, no significant difference between the two tumor entities with regard to the final activity concentration (*p* = 0.142) was observed.

Only low levels of radioactive signal were detectable in the brains of mice with initial activity concentrations of (1.95 ± 0.57) %IA/mL (1h) decreasing to (1.00 ± 0.30) %IA/mL after 24 h.

A comparison between the two tumor entities of both xenograft models mouse tumors presented significantly higher activity concentrations (*p*_TZMbl_ < 0.001; *p*_PC-3_ < 0.001) at 24 h; however, in the brain, higher activity concentrations were found within the brains of chicken embryos (*p* < 0.001).

### 3.3. Analysis of Tumor-to-Blood Ratios over 24 h

**Figure 5:**
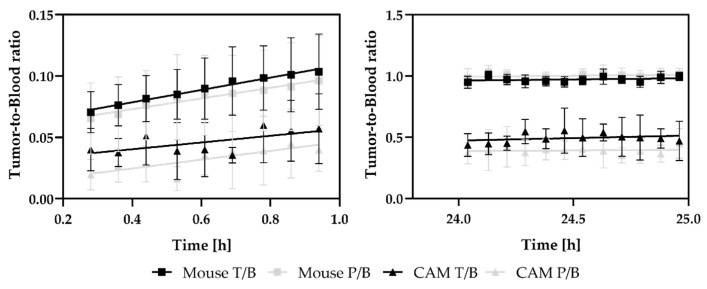
To investigate the accumulation dynamics of DFO-HSA, tumor-to-blood ratios (%) are calculated and plotted over time to derive the influx constant (K_in_) from the slope of the line. Throughout the measurement period of 24 h, no significant differences in albumin accumulation-kinetics were detectable in the TZM-bl and PC-3 tumors, both within and in comparison, between the two xenograft models. Although the overall ration in the CAM model is lower, T/B ratios demonstrate a similar kinetic.

Initially, the tumor-to-blood ratio was low in both the CAM model (T_TZM-bl_/B = 0.06 ± 0.05; T_PC-3_/B = 0.03 ± 0.03) and the mouse model (T_TZM-bl_/B = 0.10 ± 0.03; T_PC-3_/B = 0.10 ± 0.03) (Figure 5). The ratios continuously increased over the measurement period.

In the CAM model, the ratio increased with an influx constant of K_in_(TZM-bl) = (1.88 ± 0.40) (% IA/mL)/h; R^2^ = 0.83 and K_in_(PC-3) = (1.52 ± 0.35) (%IA/mL)/h; R^2^ = 0.6 finalizing at a ratio of T_TZM-bl_/B = 0.47 ± 0.16% and T_PC-3_/B = 0.42 ± 0.15% after 25 h p.i., respectively. There were no differences between the two tumor entities detected in either the final ratio (*p* = 0.485) or the slope of the accumulation kinetics (p_1h_ = 0.677; p_24h_ = 0.677).

For the SCID mouse model, tumor accumulation kinetics with an influx constant K_in_(TZM-bl) = (3.70 ± 0.07) (%IA/mL)/h, R^2^ = 0.7 and K_in_(PC-3) = (3.85 ± 0.17) (%IA/mL)/h, R^2^ = 0.8 were calculated, resulting in a tumor-to-blood ratio after 25 h of T_TZM-bl_/B = 1.00 ± 0.03 % and T_PC-3_/B = 1.02 ± 0.04 %. No statistically significant differences were observed for the 24 h ratio (*p* = 0.686) and in the slope of accumulation (*p*_1h_ = 0.814; *p*_24h_ = 0.950).

### 3.4. In Vivo and Ex Vivo Biodistribution Analysis

**Figure 6:**
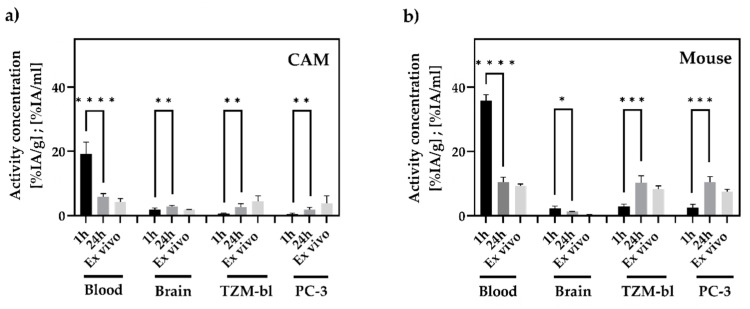
Ex vivo and in vivo biodistribution analysis (%IA/g; %IA/mL) of blood, brain, and tumors after 1 and 24 h p.i. A significantly higher amount of zirconium-89 activity in both tumor entities of both model systems after 24 h p.i. was demonstrated using PET. Significantly less activity was measured in the blood samples of both models after 24 h. While the activity concentration in the brain increased in the CAM model (**a**) after 24 h, there was a decreased observed for the mouse model (**b**). No statistically significant differences were detected between all measured in vivo activity concentrations and the corresponding ex vivo activity concentrations after 24 h. Significance was highlighted by asterisks (* = *p* ≤ 0.05; ** = *p* ≤ 0.01; *** = *p* ≤ 0.005; **** *p* ≤ 0.0001 (GraphPad Prism, unpaired *t*-test).

In the CAM model, activity concentrations using γ-counter (Figure 6, Table S8) for the blood pool of (4.29 ± 0.89) %IA/g, for the TZM-bl tumors of (4.50 ± 1.47) %IA/g, and for the PC-3 tumors of (3.79 ± 2.11) %IA/g were detected. No significant differences between the activity accumulation of the TZM-bl and PC-3 tumors were determined (*p* = 0.548).

For SCID mice, an activity concentration in the blood pool of (9.36 ± 0.45) %IA/g was detected. Activity concentrations for the tumor xenografts were determined with (7.26 ± 0.73) %IA/g for TZM-bl and (8.09 ± 0.86) %IA/g for PC-3. No relevant differences between the tumors were identified (*p* = 0.343).

The determined activity concentration in the avian brain was, on average, (1.37 ± 0.80) %IA/g, while, in the murine brains, an activity concentration of (0.31 ± 0.04) %IA/g was detected. Comparing the data of the in vivo biodistribution analysis with the results of the ex vivo analysis, significant differences were only found for the mouse (*p* = 0.029) and avian brains (*p* = 0.016). 

The in vivo and ex vivo biodistribution data are summarized in Figure 6.

### 3.5. Immunohistochemical Analysis, H&E Staining and Digital Autoradiography

**Figure 7:**
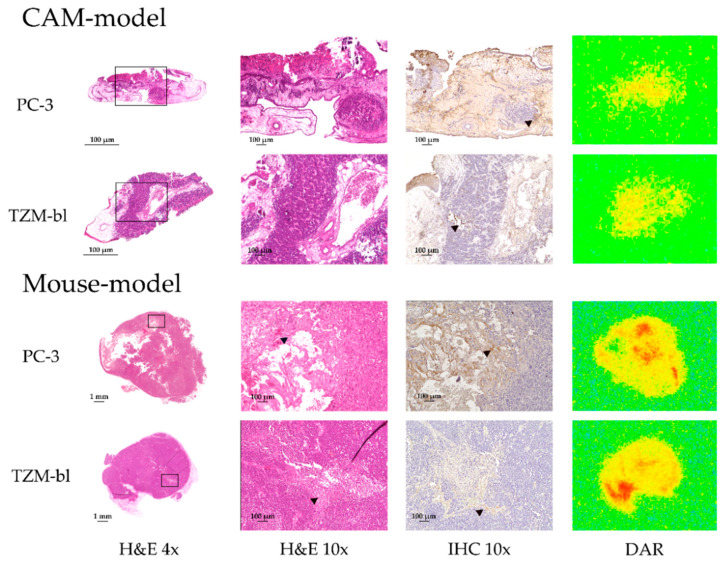
Microscopic and DAR images of histological sections of PC-3 and TZM-bl tumors of both xenograft models: In the H&E images, obvious necrotic areas (black arrow) were observable in the tumors from the mouse model. In the subsequent IHC sections, [^89^Zr]Zr-DFO-HSA accumulation was clearly visible at the same locations (brown color precipitate, black arrows). The areas stained in the IHC correspond to the regions with strong radioactivity enrichment in the DAR (coded in red). Similar areas were not noted in the tumors of the CAM model, having a more homogeneous distribution of zirconium-89 activity and HSA. In general, IHC could verify the accumulation of [^89^Zr]Zr-DFO-HSA due to the EPR effect.

By H&E staining of PC-3 and TZM-bl tumors from SCID mice, a more heterogeneous structure was demonstrated, as for the CAM model (Figure 7). In both tumor entities, larger areas were conspicuous by a softened morphology in their respective H&E staining, including strongly eosinophilic connective tissue strands and absent, blue-stained cell nuclei. These areas corresponded to colliquative tumor necrosis and were also notable upon qualitative assessment in DAR and IHC with a more intense signal of zirconium-89 activity and more intense staining of HSA compared to areas with vital tumor cells.

In contrast, the appearance in the TZM-bl and PC-3 tumors of the CAM model was more homogeneous in both digital autoradiography and H&E staining. With H&E staining, a uniform distribution of viable tumor cells with well-stained blue nuclei was observed, and no larger areas of tumor necrosis were found. Additionally, by DAR, no areas with a localized higher signal of zirconium-89 were observed.

## 4. Discussion

In this study, we demonstrated that in the CAM model, accumulation and retention of an EPR-dependent macromolecule in transplanted xenograft tumors using multimodal PET and MR imaging exhibited kinetics comparable to those in the standard mouse xenograft model. This highlights the potential of the CAM model as an alternative to animal models based on 3Rs principles for labeled macromolecules.

### 4.1. Radiolabeling and In Vitro Studies

The radioisotope zirconium-89 with a half-life of 78.4 h, allows for monitoring the biodistribution of HSA for several days by repeated PET imaging. In addition, radiolabeling can be performed with the specific chelator DFO at room temperature and pH 7, which are gentle conditions for proteins.

Successful radiolabeling of DFO albumin was demonstrated and stability was confirmed both in vitro and in vivo (Figure 1b). Thereby, it was decisive that the distribution and accumulation of free zirconium in vivo is fundamentally different from the distribution of albumin-bound zirconium, as free zirconium-89 accumulates particularly in the joints, whereas split-off [^89^Zr]Zr-DFO is excreted via the kidney [28,29], which we could not detect in our study

For BSA, it has been published that albumin is internalized via macropinocytosis in MIA PaCa cells, while for HSA, it was demonstrated that albumin is internalized via clathrin-independent, caveolin-mediated endocytosis in PBMCs and PMNs [30,31]. HSA has not been internalized in various tumor cell lines [32,33]. Our in vitro studies also confirmed that there is no internalization of HSA into the tumor cell lines TZM-bl and PC-3, as no noteworthy internalization or cell surface binding of [^89^Zr]Zr-DFO-has could be determined (Figure 1c,d). A small fraction of the administered radioactivity that appeared to be bound to the cell surface and internalized was detected to the same extent also in the experimental setup without cells (Figure S1). Consequently, uptake of labeled HSA is not significantly affected by active tumor uptake, and radiotracer accumulation is mainly caused by the passive aspects of the EPR effect.

### 4.2. In Vivo Biodistribution in CAM and Mouse Model

In general, the biodistribution of the radioligand was accurately visualized by PET imaging in both CAM and mouse xenograft models. Comparing the total activity concentration of the two xenograft models, a significant decrease over time was evident only in the mouse model, unlike in the chicken model, in which the value remained constant, as expected. This is due to the fact that the chicken egg is a functionally closed system and excreted substance would accumulate in the allantoic fluid [34,35,36,37]. In the mouse, serum albumin is excreted via the excretory organs such as the kidney and liver with an estimated half-life of around 2 days [38].

As expected, the lowest activity concentration was observed for the brains in the two xenograft models, with a slight increase in the chicken embryo model after 24 h (Figure 2), which was confirmed by the ex vivo results. The permeability of the blood-brain barrier (BBB) of chicken embryos has been investigated in recent studies. It was demonstrated that the barrier is permeable to molecules larger than 40 kDa until EDD14 [39]. The results of our study support these findings, as little to no albumin was detected to cross the BBB.

### 4.3. In Vivo Tumor Accumulation in CAM and Mouse Model

Both TZM-bl and PC-3 tumors were successfully visualized after 24 h for both xenograft models using PET (Figure 2 and Figure 3). The quantitative evaluation based on the time-activity curves and the data obtained by ex vivo analysis was congruent.

However, the accumulated activity concentration based on PET evaluation was significantly higher in mouse model xenografts than in the CAM xenografts (Figure 4). This result was further confirmed in the ex vivo studies. Importantly, the accumulation dynamics of labeled albumin from the blood pool into tumors revealed no relevant differences between TZM-bl and PC-3 tumors for either in vivo model (Figure 5). It was also evident that for the CAM model, the ex vivo data yielded higher results than the PET data, whereas the tendency in the mouse model was rather the opposite (Figure 6). Here the partial volume effect (PVE) might be relevant causing an underestimation of the activity concentration in the smaller tumor structures of the CAM-model [40,41,42].

After 24 h, the activity concentrations of blood and TZM-bl and PC-3 tumors equalized in the mouse model, although PET images suggested that accumulation was regionally higher in the tumors. However, other areas of the tumors exhibited low to moderate tracer accumulation. Consequently, the activity concentration averaged on the entire tumor volume was similar to that of the blood pool (heart). This effect was not observed in the CAM model, as the tracer accumulation appeared to be more homogeneous. The feasible impacts on the varying dynamics, such as intratumoral blood flow or the degree of vascularization, were not investigated in this study. Further MRI investigation using contrast agents in order to assess intratumoral perfusion would be beneficial. However, tumor size appeared to be a determining factor, as it directly correlates with the intratumoral necrotic fraction [43]. Tumor weight and volume reach an average 10-fold higher extent in mice (Table S9). H&E staining depicted a much more heterogeneous pattern in mouse tumors compared to the chicken embryo tumors (Figure 7). In particular, based on the qualitatively evaluated histologic sections, for the mouse tumors a much higher proportion of necrotic fraction was observed, which is known to increase with tumor volume [43]. The necrosis fractions of mouse TZM-bl and PC-3 tumors were congruent in DAR and IHC with comparatively higher and more intense tracer concentrations. Evidently, the radiotracer accumulated primarily in necrotic areas. However, it should be noted that no blood flow is detectable in the necrotic areas of the tumors [10] as tumor necrosis is the result of a chronic hypoxic environment due to a severing from the blood supply [44,45]. Within the necrotic areas, the extracellular compartment is significantly larger compared to areas with densely growing tumor cell tissue. It has been demonstrated that the radiolabeled albumin primarily accumulates passively in the extracellular space (Figure 7). These results were congruent with the results of the internalization assays, where no uptake into tumor cells could be detected (Figure 1c). Therefore, areas of tumor necrosis appeared with significantly higher concentrations, although the passive distribution in the extracellular compartment could be considered more homogeneous. No extensive necrotic areas were observed in the TZM-bl and PC-3 tumors of the CAM model, and, in agreement, the activity concentration was also more homogeneously distributed in the tumor. Successful accumulation of radiolabeled HSA in the interstitial space of the analyzed tumors was possible because of the deficient endothelia of the blood vessels, which can be considered as a model for barrier defects.

### 4.4. Limitations

There are, of course, physiological and developmental time-related differences between chicken embryo and SCID mouse. Particularly relevant to the analyses were tumor weight and volume, which are 10-fold lower in the CAM model than in the mouse model (Table S9). Tumor growth is affected by the number of cells used and the maximum possible incubation period, which were 7 days in the CAM model. In comparison, tumors in the mouse were grown over a period of 14 days. The use of a larger cell number could be expected to result in larger CAM tumors. However, in tumors derived from larger tumor cell concentrations we frequently observed bleed-in into the tumors. Increased silicone rings could compensate for the higher cell concentration, but this will complicate placing two different tumor xenografts on one egg while leaving space for injection. Tumor size and volume not only determine the absolute amount of recorded activity, but also the relative effect of irradiating signal from neighboring blood vessels in the PET measurements. It is well known that the partial volume effect (PVE) has a greater influence on small structures [41]. Appropriate partial volume corrections are challenging but could pay off with more extensive use of the CAM model. With the number of mice and eggs used, the first comparative evaluation of the accumulation and distribution of albumin in the chicken embryo model compared to the mice could be performed. With a larger number of chicken eggs in future experiments, the results could be verified, described limitation could be improved and more precise data might be obtained.

## 5. Conclusions

In conclusion, the effects of the EPR effect and importantly the accumulation process of the radiotracer [^89^Zr]Zr-DFO-HSA in the interstitium of TZM-bl and PC-3 tumors was detectable in both xenograft models and progressed similarly in both models. The TZM-bl and PC-3 tumors of the chicken embryos were smaller and more homogeneously structured than their counterparts in the mouse model, thus being less effected by necrotic areas. Therefore, the CAM model represents a possible alternative not only regarding aspects to study the accumulation of EPR-dependent macromolecules but also in terms of the 3Rs principles. Especially in the initial phase of pharmaceutical development, the CAM model can reduce the number of animals needed. Furthermore, the CAM model in combination with HSA seems to be well suited for the detection of alterations in the endothelial barrier of blood vessels. Both, the pathologically disrupted blood–tumor barriers and the physiologically intact blood–brain barriers could be evaluated. This potentially enables studies on barrier disfunctions also in the respect of trauma research.

## Data Availability

The used data, additional to those in the supplement, are available from the corresponding author on reasonable request.

## Supplementary Materials

The following supporting information can be downloaded at: https://www.mdpi.com/article/10.3390/cancers15041126/s1, Figure S1: Cell surface binding and internalization of [^89^Zr]Zr-DFO-HSA and [^89^Zr]; Figure S2: [^89^Zr]Zr-DFO-HSA distribution illustrated in the avian heart; Figure S3: Biodistribution of [^89^Zr]Zr-DFO-HSA in the CAM model 24 h p.i.; Table S1: Cell surface binding and internalization of 89-zirkonium and [^89^Zr]Zr-DFO-HSA in TZM-bl and PC-3 cells; Table S2: Cell surface binding and internalization of 89-zirkonium and [^89^Zr]Zr-DFO-HSA without TZM-bl and PC-3 cells; Table S3: Total applied activity for internalization of DFO-HSA; Table S4: Data based on activity concentrations 1 h post-injection calculated with PMOD in the murine model (%IA/mL); Table S5: Data based on activity concentrations 24 h post-injection calculated with PMOD in the murine model in (%IA/mL); Table S6: Data based on activity concentrations 1 h post-injection calculated with PMOD in the CAM model in (%IA/mL); Table S7: Data based on activity concentrations 24 h post-injection calculated with PMOD in the CAM model (*%IA/mL); Table S8: Final activity measured with PET and γ-counter (%IA/mL) and (%IA/mg). Table S9. Overview of tumor weights [g].
