# Supplementary material for: Evaluation of the EPR Effect in the CAM-Model by Molecular Imaging with MRI and PET Using 89Zr-Labeled HSA
Source: Cancers (Basel). 2023 Feb 9;15(4):1126. doi: 10.3390/cancers15041126 (PMC9954539; doi:10.3390/cancers15041126)
Supplement: Supplementary file 1 [file cancers-15-01126-s001.zip › cancers-2185780-supplementary.pdf]

Evaluation of the EPR Effect in the CAM-Model by Molecular Imaging with MRI and PET Using ^{89}Zr-Labeled HSA

Colmar Hilbrig [1,*,†], Jessica Löffler [1,2,†], Gabriel Fischer [1], Ellen Scheidhauer [1], Christoph Solbach [1], Markus Huber-Lang [3], Ambros J. Beer [1], Volker Rasche [2,4] and Gordon Winter [1,*]

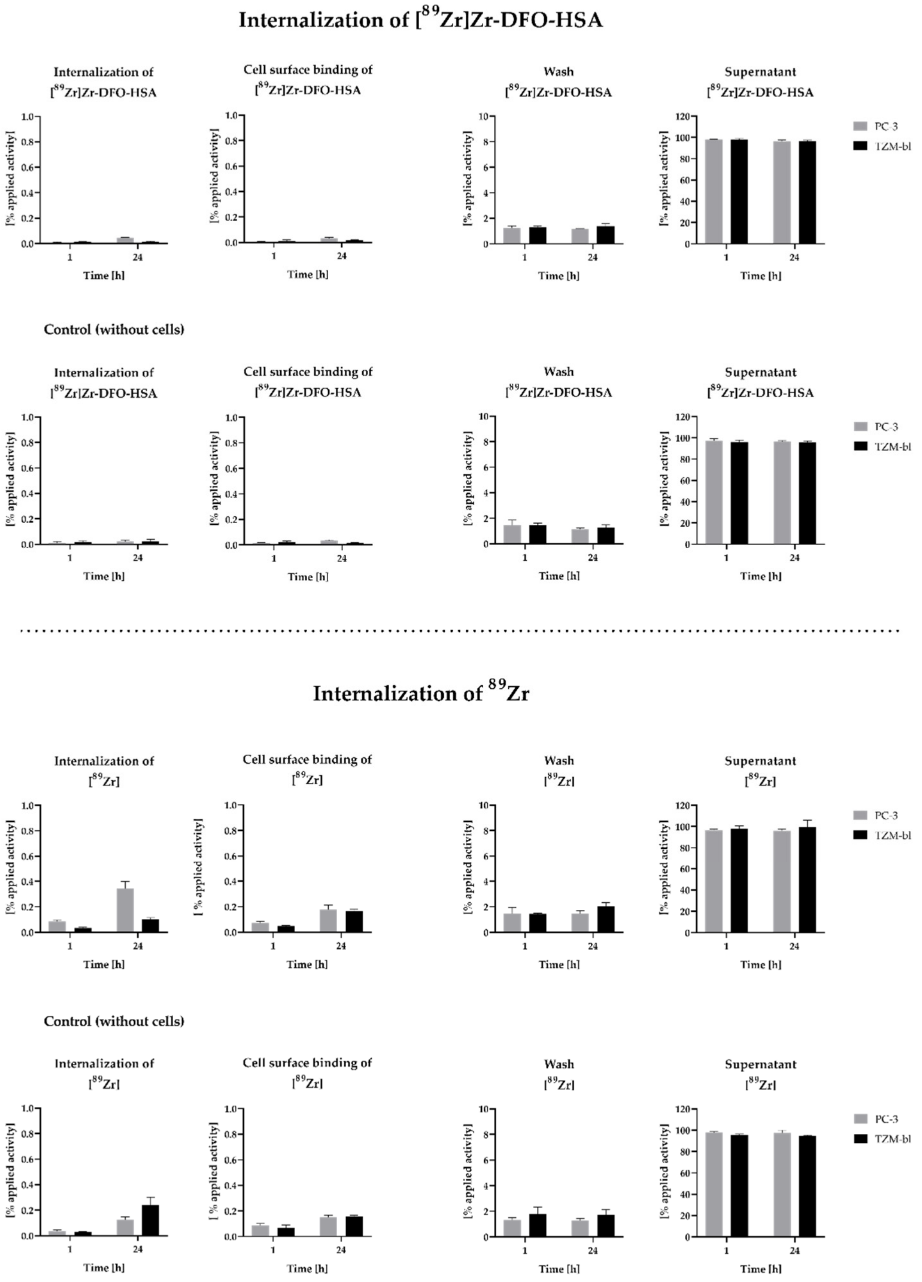

Figure S1. Cell surface binding and internalization of [^{89}Zr]Zr-DFO-HSA and zirconium-89 to PC-3 and TZM-bl cells. Binding to the cells as well as internalization of [^{89}Zr]Zr-DFO-HSA into the cells was negligible after 1 h and 24 h. Binding of zirconium-89 to PC-3 cells increased from (0.07 ± 0.01) % to (0.18 ± 0.03) % within 24 h. Internalization of zirconium-89 into PC-3 cells also increased from (0.09 ± 0.01) % to (0.35 ± 0.06) %. Binding and internalization of zirconium-89 into TZM-bl cells is also negligible.

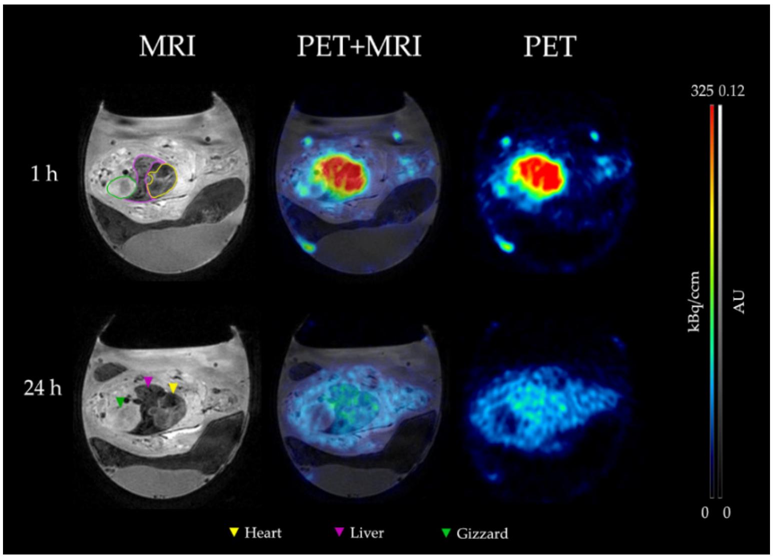

Figure S2. [^{89}Zr]Zr-DFO-HSA distribution illustrated in the avian heart. MR and PET image depict the same chicken egg as Figure 2, including a fusion image, of the CAM model injected with [^{89}Zr]Zr-DFO-HSA after 1h and 24h. In the T1-weighted Flash 3D sequence (left) the anatomy of the chick embryo was clearly visible, especially the heart (yellow arrow), the liver (magenta arrow) and gizzard (green arrow). The biodistribution of the radioligand could be observed in the PET images (right). During the first hour most activity is still in the blood pool, indicated by the pronounced signal coming from the heart region and blood vessels, which spilled over especially into the liver and gizzard. After 24 h the injected activity is distributed more homogeneously, except for the gizzard where no signal is detectable.

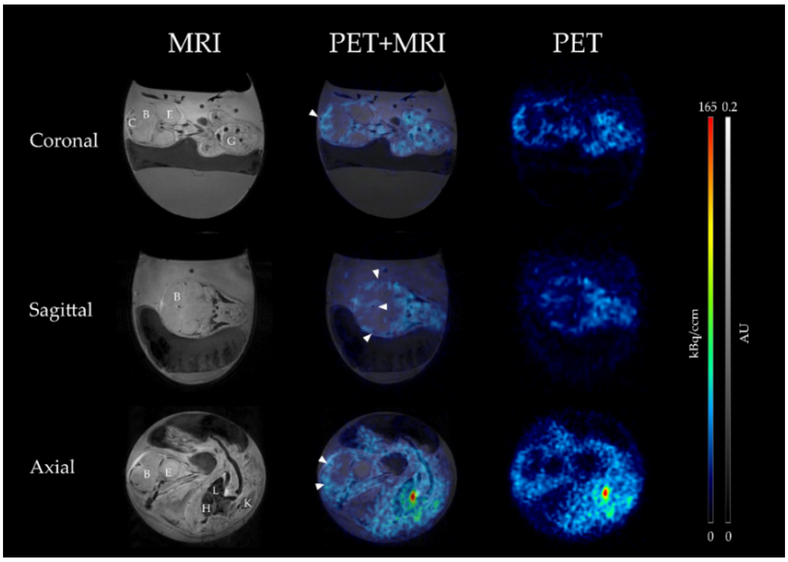

Figure S3. Biodistribution of [^{89}Zr]Zr-DFO-HSA in the CAM model 24h p.i. The anatomy of the chick embryo was acquired by a T1-weighted Flash 3D sequence (left). Brain (B), eye (E), cerebellum (C) gizzard (G), heart (H), liver (L) and kidneys (K) can be easily distinguished. A static reconstruction of the 60-min PET scan was used to demonstrate [^{89}Zr]Zr-DFO-HSA distribution (right). In the resulting fusion image (middle), the accumulated activity was assigned to the corresponding organs. In the brain or more precisely the cerebrum only a weak signal was detected. Within the brain, major activity was detected in the region of the cerebellum (C), where spillover through the major meningeal blood vessels (white arrows) influenced activity determination.

Table S1. Cell surface binding and internalisation of 89-zirkonium and [^{89}Zr]Zr-DFO-HSA in TZM-bl and PC-3 cells

	**[^{89}Zr]Zr-DFO-HSA [counts/min]**				**[^{89}Zr] [counts/min]**			
	PC-3		TZM-bl		PC-3		TZM-bl	
	1h	24h	1h	24h	1h	24h	1h	24h
**Internalization**	16.08	193.20	71.73	27.11	134.28	1667.89	210.09	1667.89
	58.92	198.70	60.98	81.29	225.64	1872.36	226.36	1872.36
	16.09	225.59	61.04	65.03	247.24	2287.05	172.52	2287.05
**Cell surface binding**	14.63	171.80	55.02	94.87	370.90	832.45	225.75	1021.98
	41.92	164.10	38.52	56.03	370.92	1010.85	303.00	854.30
	23.46	116.58	93.44	72.73	505.03	1215.37	284.37	899.17
**Wash**	6401.64	5080.15	5791.61	7169.31	7520.82	7162.38	8558.56	12382.80
	5037.47	5248.11	6430.04	6091.21	6290.92	9318.18	8019.28	9815.23
	5738.82	5302.26	5526.61	5635.48	11473.82	8831.48	8036.39	12732.21
**Supernatant**	444835.76	436360.00	442973.16	435595.30	550692.12	552913.24	570381.22	606104.53
	445354.93	439882.93	447263.34	440667.16	553057.05	548271.65	556650.13	537178.28
	441894.00	429355.27	440310.85	435960.85	542723.85	534163.19	539303.24	548687.20

Table S2. Cell surface binding and internalisation of 89-zirkonium and [^{89}Zr]Zr-DFO-HSA without TZM-bl and PC-3 cells

	**[^{89}Zr]Zr-DFO-HSA [counts/min]**				**[^{89}Zr] [counts/min]**			
	PC-3		TZM-bl		PC-3		TZM-bl	
	1h	24h	1h	24h	1h	24h	1h	24h
**Internalization**	32.19	69.19	120.19	168.14	134.28	610.77	165.09	1773.15
	30.10	139.37	104.11	27.13	225.64	844.07	145.64	1128.65
	112.64	106.90	34.10	108.50	247.24	695.73	188.74	1174.92
**Cell surface binding**	433.80	122.09	58.36	94.94	433.80	752.25	358.15	838.33
	448.28	164.22	93.55	59.51	448.28	896.22	297.60	915.95
	587.72	177.46	143.01	72.84	587.72	930.94	518.20	933.12
**Wash**	7469.84	5694.39	7464.86	5150.79	6597.79	8131.20	13622.46	8918.79
	7854.41	5017.94	6037.03	6833.93	7059.29	7456.57	8055.45	8263.64
	4767.97	4945.10	6241.98	4981.29	8770.71	6487.20	8772.99	12418.06
**Supernatant**	448453.29	440591.21	441635.23	429459.73	554725.24	550182.18	536271.83	534432.41
	438750.89	436833.54	437061.56	439822.29	560035.58	568628.55	542069.80	541495.11
	431353.11	435556.37	425576.92	431422.67	554649.78	540151.46	546425.30	531592.02

Table S3. Total applied activity for internalization of DFO-HSA

**[^{89}Zr]Zr-DFO-HSA**	**[^{89}Zr]**
459975.86	459975.86
449032.89	449032.89
447401.43	447401.43

Table S4. Data based on activity concentrations 1 h post injection calculated with PMOD in the murine model [%IA/mL]

Time	Blood				TZM-bl				PC-3				Brain				Mouse			
0.0028	0.2789	1.7384	0.4287	1.7547	0.4374	1.0596	1.2084	0.8960	0.3869	0.6886	1.0122	0.6648	0.1152	0.5050	0.1202	1.3922	0.4138	0.9484	0.9932	0.8552
0.0083	0.7020	1.3517	0.6477	1.0437	0.5624	0.7180	0.9048	0.6636	0.4994	0.7540	1.0265	0.7528	0.1341	0.3253	0.2431	1.2353	0.4441	0.9071	0.9834	0.8555
0.0139	36.3397	26.9537	19.7676	1.5651	1.4803	1.3930	1.4462	0.8972	1.0604	2.1515	1.1864	0.6799	1.2263	3.6940	1.4418	0.4544	3.9731	5.2926	4.6051	0.9136
0.0194	47.9407	37.2247	31.4775	37.5193	1.7359	3.1037	2.5442	1.7918	1.2582	3.2644	2.5741	2.1362	3.0658	4.7786	4.0362	3.7631	6.1016	8.6478	8.5682	5.3094
0.0250	44.3291	35.1251	36.6909	34.1978	1.6493	3.2093	2.1843	2.2125	1.8856	3.3544	2.1023	2.6017	3.7849	2.4484	4.5752	3.2526	6.5175	8.2234	9.0170	7.0563
0.0306	39.6616	34.4215	33.4822	45.1541	1.8350	3.0523	2.6797	3.4529	2.2529	3.1342	2.9843	3.6880	1.2931	3.7249	3.5334	3.6804	6.4922	8.1572	9.8485	8.2446
0.0417	40.6767	30.5557	34.4411	41.8183	1.6596	1.8227	2.0612	1.9174	1.3551	2.9210	1.6835	2.4558	0.9312	2.8084	3.0236	3.8959	5.5349	6.7867	8.0005	6.8656
0.0583	39.4859	31.4994	33.7685	40.8377	1.7715	2.3933	2.0560	2.3712	1.3294	2.6451	1.6929	2.3217	2.9050	4.0880	1.9086	3.5861	5.5758	6.6670	7.9831	6.7964
0.0750	38.6593	28.8763	29.9448	40.9088	1.9101	2.4914	2.2336	2.5751	1.4259	2.6428	1.7709	2.8911	3.9239	2.8058	2.4441	3.4799	5.4928	6.7532	7.8528	6.8173
0.0917	39.2270	29.9018	33.2477	38.4493	1.8659	2.4724	2.4703	2.4688	1.5830	3.5642	2.0582	2.6328	3.3885	4.1046	2.2751	3.5412	5.4782	6.6739	8.5410	6.6525
0.1083	41.0290	29.4685	32.7350	38.0654	1.9408	2.6914	2.2587	2.9491	1.5765	3.5681	2.0754	2.7613	1.2868	2.3422	3.0220	3.4193	5.4844	6.6474	8.2646	6.8044
0.1250	41.3012	29.3097	31.5089	38.8230	1.8868	2.8017	2.2114	2.9677	1.3365	2.5377	1.8954	2.8605	1.6895	1.9596	3.5239	3.4426	5.5177	6.6454	8.0120	6.8020
0.1417	41.1778	28.7458	32.6819	40.3346	1.9381	2.3211	2.5497	3.3256	1.5080	2.8709	1.9239	2.8349	0.5864	1.3727	1.8936	3.3820	5.5190	6.6464	8.0072	7.1220
0.1917	39.2213	29.2184	30.7022	38.8234	1.8134	2.2708	1.9551	2.7934	1.3443	2.5444	1.5364	2.7210	2.2142	2.2843	1.1946	1.9836	5.1578	6.1480	7.1514	6.2429
0.2750	39.6610	28.6938	31.2658	38.9371	1.9750	2.3883	2.0045	3.3132	1.4617	2.9659	1.5765	2.7631	1.8959	2.5011	1.0038	2.3858	5.1624	6.1178	7.1954	6.1782
0.3583	39.6920	29.0917	32.1323	38.0357	2.2957	2.4586	2.1727	3.6304	1.4649	3.1257	1.7531	2.9172	1.6306	1.7998	1.9707	1.8983	5.1302	6.0997	7.1628	6.1967
0.4417	37.2405	29.0297	32.0940	37.0189	2.3909	2.1620	2.5697	3.9986	1.7972	3.1795	1.8732	3.3022	2.5602	1.7399	1.3485	2.1402	5.1323	6.0728	7.2042	6.1394
0.5250	38.3806	28.8597	31.3724	36.9492	2.5580	2.4640	2.3676	4.1847	1.8141	3.5886	1.9692	3.5972	2.8415	1.3993	2.0050	1.8970	5.1201	6.0698	7.0959	6.1239
0.6083	36.8762	29.1378	31.7093	36.6224	2.5202	2.5948	2.4406	4.5828	1.9492	3.5213	1.9430	3.7428	1.3398	2.4262	2.1398	2.4156	5.0910	6.0696	7.0862	6.0938
0.6917	37.1765	28.2184	31.3864	36.1929	2.6218	2.7539	2.5533	4.8559	2.0544	3.2919	1.9835	3.9161	2.6296	1.9936	2.3962	2.1862	5.0436	6.0235	7.1098	6.1928
0.7750	36.2967	28.4346	31.0495	35.8105	2.8009	2.7345	2.6231	4.8652	2.0975	3.5134	1.9452	3.8909	1.6146	2.1069	2.0033	2.1074	5.0554	6.0129	7.0908	6.0886
0.8583	36.6617	28.7833	30.6463	35.2432	2.7066	2.7191	2.8178	5.0842	2.1590	3.9095	2.0187	3.6961	2.0063	2.1933	2.0519	1.9527	5.0610	6.0246	7.0461	6.0321
0.9417	37.3545	28.1575	30.7297	35.4023	2.8009	2.8046	2.8545	5.2001	2.2832	3.9223	2.1975	4.0140	2.0177	1.8471	2.5729	1.9891	5.0288	5.9590	7.0630	6.0439

Table S5. Data based on activity concentrations 24 h post injection calculated with PMOD in the murine model in [%IA/mL]

Time	Blood				TZM-bl				PC-3				Brain				Mouse			
24.0417	9.5504	9.1108	8.0258	10.0459	9.2386	8.9684	7.7783	8.8028	9.6330	9.0904	8.4912	8.9125	1.4387	1.0930	0.7206	0.8409	4.7042	4.9190	3.4567	5.3161
24.1250	9.5638	8.9341	7.5959	9.0174	9.2491	9.3061	7.8210	8.8897	9.6396	8.9644	8.4873	8.9524	1.1157	0.4992	0.8575	0.9436	4.6658	4.9228	3.4473	5.2696
24.2083	9.7019	9.5529	8.3711	8.5967	9.1596	9.2654	7.8773	8.8749	9.5360	9.2680	8.7152	9.0168	1.0458	1.1000	0.6865	1.2599	4.6788	4.8805	3.4653	5.1854
24.2917	10.1464	9.0350	8.7784	9.5182	9.5240	9.3279	8.0091	8.9819	9.5159	9.1748	8.6702	8.9609	1.5658	1.2901	1.0363	1.0344	4.6568	4.9049	3.4441	5.1605
24.3750	10.1564	9.2844	8.5334	8.9997	9.3613	9.2317	7.9091	8.8986	9.7120	8.9366	8.7732	9.2166	1.2507	0.8322	0.5885	0.9952	4.6462	4.8753	3.4289	5.2342
24.4583	9.9646	9.5577	9.5040	9.3021	9.5645	9.3133	8.4785	9.1797	9.6686	9.4227	9.0395	8.9766	1.6235	1.4843	0.7225	0.8952	4.6598	4.9064	3.5021	5.2257
24.5417	10.1939	8.8828	8.5455	8.9315	9.5012	9.0099	8.0592	8.6745	9.5746	9.1663	8.9009	9.1672	0.8911	0.8302	0.4885	0.7948	4.6400	4.8904	3.4346	5.2381
24.6250	10.2110	8.8850	8.1965	8.3267	9.3983	9.2978	7.9328	8.7422	9.6983	9.0100	8.7661	9.0269	0.9846	0.5645	1.3675	0.8331	4.6443	4.8624	3.4329	5.2086
24.7083	9.6166	9.3103	8.0624	9.7859	9.2842	9.1228	8.1463	9.1340	9.6039	9.2959	8.7575	9.2141	1.6359	0.7239	1.3503	1.2273	4.6276	4.8755	3.4363	5.2109
24.7917	10.2108	9.3427	8.2726	9.0167	9.0561	9.2056	7.9802	8.8065	9.6092	9.1454	8.7860	9.0171	1.0759	0.5720	0.8358	1.1423	4.6200	4.8351	3.4424	5.2263
24.8750	9.9052	9.2681	8.1427	8.7907	9.1502	9.1753	8.1062	9.2019	9.5860	9.0787	8.7069	9.1474	1.1958	0.9136	0.6937	0.9258	4.6145	4.8633	3.3839	5.2465
24.9583	9.9596	9.1962	8.2058	8.7740	9.4368	9.2607	8.3180	8.9434	9.6351	9.1145	8.7463	9.1550	1.0371	1.2879	1.3576	0.5469	4.6152	4.8858	3.4120	5.1715

Table S6. Data based on activity concentrations 1 h post injection calculated with PMOD in the CAM model in [%IA/mL]

Time	Blood					TZM-bl					PC-3					Brain					Chicken embryo				
0.0028	0.1827	1.0306	0.1487	6.7424	1.2815	0.0558	0.4464	0.1151	0.1286	0.1803	0.1839	0.0926	0.2155	0.2819	0.3427	0.1726	0.3085	0.0841	0.1392	0.1552	0.1611	0.3937	0.2573	0.6133	0.3680
0.0083	31.2287	0.6249	27.9243	37.5435	91.6061	0.4500	0.2490	0.3701	0.7072	0.2868	0.1307	0.0659	0.4041	1.0863	0.4041	0.5252	0.4700	0.2361	0.6699	1.2317	2.1443	0.3836	1.9984	2.3911	3.5902
0.0139	37.6885	1.4012	30.2145	21.2316	89.7570	0.7291	1.4092	2.7894	0.6971	0.9161	0.8258	0.1625	1.2855	0.4859	1.1415	2.4572	0.3516	0.5840	1.1887	3.7676	3.2065	0.6181	2.9210	2.6936	4.1477
0.0194	24.8946	19.9451	26.0299	17.4056	54.5703	0.3538	2.4584	0.7878	0.7601	1.0440	0.1803	1.5287	0.0812	0.2709	0.7991	2.1104	1.5818	0.8582	1.2192	3.5622	3.1395	2.2500	2.7873	2.7398	3.7948
0.0250	22.2918	15.2415	23.7558	17.1420	40.3821	0.3251	2.5321	2.2582	0.7296	0.1309	0.2393	1.0871	0.2918	0.8547	0.4286	2.3957	2.5694	0.9846	1.1485	2.6574	3.2506	2.6383	2.7979	2.7750	3.7151
0.0306	21.7623	19.0532	20.7743	18.6250	37.2338	0.6278	2.8534	1.8376	1.0992	0.1783	0.2622	1.4015	0.9051	0.1823	0.7499	2.3707	2.1953	1.1411	1.1771	2.1646	3.3268	2.6555	2.8031	2.7733	3.6901
0.0417	21.7957	18.8358	19.9128	18.3118	33.6914	0.7518	1.5225	0.9525	0.3314	0.5457	0.2524	0.0760	0.4138	0.6019	0.6634	2.2336	1.3218	0.7607	1.2258	1.6927	3.0518	2.0960	2.3944	2.5788	3.3989
0.0583	23.0542	18.8422	22.4588	18.2584	30.2957	0.4738	1.0112	1.7255	0.6304	0.8544	0.2345	0.5590	0.4574	0.5878	0.9499	2.0260	1.4860	0.8277	1.2344	1.9380	3.0780	2.1150	2.3984	2.5766	3.3368
0.0750	22.1900	21.2495	20.2431	18.0814	27.9856	0.7485	1.2217	2.0232	0.1235	0.4906	0.1030	0.0533	0.3965	0.6527	1.0434	2.0186	1.7315	0.9512	1.2327	2.1639	3.0754	2.4150	2.3336	2.5591	3.3374
0.0917	20.8791	22.1360	16.7512	18.3182	27.2122	0.5114	1.9194	1.6851	0.9419	0.7606	0.0278	0.1640	0.4699	0.5717	0.5846	2.1644	1.6734	1.0388	1.1581	2.0607	3.0722	2.5085	2.3291	2.5675	3.3054
0.1083	19.8635	21.5090	18.1102	17.8369	26.4727	1.3378	2.9130	0.9051	0.5698	0.3062	0.1912	0.5416	0.4042	0.3738	1.3701	2.0938	1.7753	0.9190	1.1633	2.0754	3.0696	2.7094	2.2345	2.5720	3.2542
0.1250	22.1182	21.9876	20.9398	17.3651	26.0910	0.6345	1.4616	2.1354	0.5727	0.5823	0.0097	0.5765	0.8656	0.5335	1.0402	2.0017	1.6216	0.9603	1.1880	2.1809	3.0634	2.7014	2.1966	2.5636	3.3382
0.1417	21.2895	22.6109	13.7944	18.2631	24.8536	0.1173	1.4282	1.2396	0.2749	0.9662	0.2138	0.7628	0.0234	0.6384	0.6017	2.0538	2.0802	0.9658	1.1667	2.0675	3.0784	2.7227	2.1481	2.6105	3.2977
0.1917	21.0703	21.9960	18.2712	17.1076	24.4256	0.2507	1.2076	1.4471	0.5054	0.5658	0.0372	0.2287	0.1362	0.4871	0.4780	1.9201	1.7954	0.8830	1.1193	2.1087	2.9302	2.5323	2.0043	2.4886	3.2189
0.2750	20.4389	22.6011	17.4093	16.6337	23.6723	0.4004	0.9917	1.1362	0.6974	0.6981	0.1650	0.2554	0.2559	0.6335	0.6259	1.9741	1.8156	0.8441	1.0430	2.0141	2.8628	2.5604	1.9562	2.4791	3.1959
0.3583	19.0180	22.5210	16.8636	16.0450	23.3360	0.4708	1.1786	0.7692	0.5927	0.6665	0.2589	0.3529	0.4046	0.6308	1.0031	2.0123	1.7590	0.8278	1.1177	2.0357	2.7697	2.5662	1.9315	2.4680	3.1695
0.4417	19.1683	22.2039	15.8735	14.5514	23.4191	0.8468	1.6402	1.2216	0.4507	0.6612	0.4272	0.1519	0.6824	0.5722	0.7130	2.0513	1.7352	0.8112	1.1731	1.9444	2.7721	2.5634	1.8899	2.4447	3.1730
0.5250	19.3335	21.2603	15.3637	14.0008	23.4028	0.3828	1.3823	0.9683	0.3933	0.4330	0.2652	0.0654	0.2908	0.4282	0.3676	2.0114	1.8491	0.8037	1.1061	1.9386	2.7443	2.5621	1.8816	2.4413	3.1489
0.6083	18.9839	21.1867	13.7758	13.6772	22.7590	0.7716	1.3915	0.7884	0.1776	0.5437	0.3601	0.5712	0.8182	0.6147	0.6417	2.1102	1.7799	0.8317	1.0673	1.9707	2.7186	2.5461	1.8614	2.4394	3.1508
0.6917	18.2056	19.2024	13.8019	13.4839	22.6997	0.7542	0.6554	0.5777	0.4602	0.6032	0.0664	0.4618	1.0967	0.8125	0.5861	2.1561	1.8248	0.8576	1.1312	1.9855	2.6786	2.5397	1.8611	2.4439	3.1354
0.7750	18.8609	18.5730	12.8009	12.1684	20.1930	0.6671	1.2193	1.3842	0.7121	0.6417	0.0763	0.4640	1.0233	0.5285	0.8950	2.2127	1.8463	0.8038	1.4050	1.8693	2.7205	2.5037	1.8568	2.4327	3.1046
0.8583	18.3338	17.8963	12.6444	12.6727	20.9193	0.8476	1.2559	1.1480	0.3773	0.8393	0.2212	0.4577	1.0657	0.6006	1.1696	2.0323	1.5775	0.8400	1.4011	1.8704	2.7567	2.5200	1.8661	2.4389	3.1158
0.9417	17.8249	17.5156	12.6841	12.0131	19.3804	0.6376	1.3122	1.2515	0.4600	0.7293	0.5263	0.2806	0.7664	0.5372	0.9487	1.9892	1.7290	0.8573	1.4252	1.8359	2.7194	2.4966	1.8576	2.4445	3.1020

Table S7. Data based on activity concentrations 24 h post injection calculated with PMOD in the CAM model [%IA/mL]

Time	Blood					TZM-bl					PC-3					Brain					Chicken embryo				
24.0417	7.6427	4.9099	4.5106	4.8306	6.8548	2.5051	2.1816	2.6184	1.9233	2.9708	2.5362	2.5057	1.5807	1.3698	2.5281	3.3333	1.5904	1.3008	2.1344	2.2728	2.9857	2.4057	2.4628	2.9056	3.3031
24.1250	7.7838	4.9200	4.7781	4.8702	6.5502	3.6476	2.0596	2.8163	1.7463	2.6244	2.4628	3.3332	1.2117	1.5907	2.6445	3.3800	1.5390	1.2797	2.1534	2.3068	2.9784	2.4284	2.4316	2.9232	3.2944
24.2083	7.5320	4.8371	4.5711	4.8951	7.0488	3.4233	1.8728	2.2084	2.5867	2.8416	2.4310	3.4078	1.9851	1.4797	2.4457	3.2395	1.6144	1.2664	2.1615	2.3107	2.9465	2.4297	2.4339	2.9060	3.2978
24.2917	7.6370	4.6075	4.7457	4.7861	7.0234	5.3838	2.0315	2.3457	2.7040	3.7075	2.6471	2.5982	1.3551	1.6731	2.3417	3.3465	1.6506	1.2734	2.3507	2.2733	2.9727	2.4198	2.4306	2.9355	3.3012
24.3750	7.4360	4.8493	4.7864	5.0422	6.8882	4.2213	1.9053	2.7790	2.2847	3.0803	2.7251	2.6487	1.6455	2.0389	2.3525	3.3487	1.5734	1.2812	2.3040	2.3268	2.9239	2.4016	2.4274	2.9167	3.2752
24.4583	6.9076	4.9188	4.1183	4.7509	7.0005	5.7562	2.2491	2.6789	1.8857	3.0180	2.3198	2.5391	1.3630	1.8950	2.7696	3.2295	1.5622	1.1908	2.2349	2.3514	2.9206	2.3962	2.4569	2.9217	3.2859
24.5417	7.1266	5.0466	4.9489	4.9335	7.0404	5.3956	2.2954	2.2929	2.2741	2.4525	3.2961	2.3487	1.8386	1.9945	2.3371	3.2420	1.5100	1.2494	2.2673	2.2504	2.9116	2.4004	2.4483	2.9258	3.2823
24.6250	7.4581	5.0209	4.6444	4.4778	6.8360	4.7209	2.9362	2.2419	2.3628	3.2096	2.8150	3.0808	1.6522	1.0384	2.4978	3.1666	1.6536	1.2686	2.2329	2.2956	2.8712	2.3950	2.4105	2.9025	3.2760
24.7083	7.1448	4.8334	4.6226	4.9021	7.0590	5.5503	1.9814	2.3715	2.1107	2.7579	2.5488	1.6881	1.9969	1.2707	2.6177	3.2057	1.5879	1.3183	2.2864	2.3163	2.8619	2.4106	2.4225	2.9187	3.3077
24.7917	6.9383	5.0045	4.7765	4.8470	7.2260	5.4147	1.8524	2.7959	1.9678	2.5008	3.4213	1.4651	2.1379	1.3118	3.1960	3.0967	1.5988	1.3008	2.2196	2.3804	2.8188	2.3984	2.4287	2.9161	3.3042
24.8750	7.2032	4.9313	5.1698	4.8757	7.0582	4.4884	2.4705	2.3822	2.1441	3.0406	2.6250	2.0186	1.3626	1.6900	3.1657	3.0594	1.5666	1.1947	2.2211	2.3018	2.7984	2.3951	2.4328	2.9360	3.2957
24.9583	6.6246	4.5422	4.2821	4.8982	7.1570	3.9303	1.5780	2.9567	1.6860	2.6980	2.9872	2.9277	1.9532	1.7014	2.5242	2.9450	1.5391	1.2726	2.2368	2.2825	2.7870	2.4127	2.4268	2.9524	3.3013

Table S8. Final activity measured with PET and γ-counter [%IA/mL] and [%IA/mg]

PET								γ-counter							
Blood CAM	Blood Mouse	Brain CAM	Brain Mouse	PC-3 CAM	PC-3 Mouse	TZM-bl CAM	TZM-bl Mouse	Blood CAM	Blood Mouse	Brain CAM	Brain Mouse	PC-3 CAM	PC-3 Mouse	TZM-bl CAM	TZM-bl Mouse
8.321	6.940	3.219	1.345	2.576	8.090	4.187	6.992	5.454	9.980	1.820	0.350	1.510	7.723	6.600	7.536
11.868	4.742	2.740	1.312	1.575	11.793	1.408	11.881	3.334	9.120	1.740	0.280	3.750	8.343	2.690	9.735
11.161	5.294	2.442	1.192	1.588	11.674	2.339	10.736	3.623	9.530	1.930	0.370	2.620	6.676	2.980	8.066
10.400	6.946	3.046	1.334	2.615	10.203	2.905	11.347	5.265	8.640		0.240	3.340	7.253	5.320	7.835
7.200		3.216		2.904		4.257		3.767				7.730		4.910	

Table S9. Overview of tumor weights [g].

Tumors [g]	CAM model	SCID Mouse model
TZM-bl	0.031 ± 0.005	0.388 ± 0.124
PC-3	0.015 ± 0.006	0.391 ± 0.114
